# Acupuncture for perceived stress in pregnant women: an intervention study^*^

**DOI:** 10.1590/1980-220X-REEUSP-2021-0233en

**Published:** 2022-05-30

**Authors:** Nicolau da Costa, Eveliny Silva Martins, Ana Karina Bezerra Pinheiro, Paula Renata Amorim Lessa Soares, Priscila de Souza Aquino, Régia Christina Moura Barbosa Castro

**Affiliations:** 1 Universidade Federal do Ceará, Departamento de Enfermagem, Programa de Pós-Graduação em Enfermagem, Fortaleza, CE, Brazil.

**Keywords:** Pregnant Women, Psychological Distress, Acupuncture, Complementary Therapies, Obstetric Nursing, Mujeres Embarazadas, Distrés Psicológico, Acupuntura, Terapias Complementarias, Enfermería Obstétrica, Gestantes, Estresse Emocional, Acupuntura, Terapias Complementares, Enfermagem Obstétrica

## Abstract

**Objective:**

To analyze the effects of acupuncture in the treatment of perceived stress in pregnant women.

**Method:**

A before-after intervention study, carried out in a primary health unit in Fortaleza-Ceará, with 56 pregnant women. The pregnant women underwent six acupuncture sessions, with two 30-minute sessions per week. Before the first session, an instrument to collect sociodemographic, clinical, and obstetric data was applied. The Global Perceived Stress Scale (PSS10) was applied weekly to monitor the progression of stress during treatment.

**Results:**

After the intervention, there was a significant decrease in the scores of the following scale items: being upset, inability to control, nervousness, tiredness, anger, and inability to overcome stress. (p < 0.05). There was a significant increase in the score of the item control of situations (p = 0.003). There was a significant difference in the mean perceived stress of the initial session compared to the 1st, 2nd and 3rd week sessions (p < 0.001).

**Conclusion:**

The use of acupuncture to treat stress during pregnancy reduced the stress perceived by pregnant women.

## RESUMO

**Objetivo:**

Analisar os efeitos da acupuntura no tratamento de estresse percebido em gestantes.

**Método:**

Estudo de intervenção do tipo antes e depois, realizado em unidade básica de saúde de Fortaleza-Ceará, com 56 gestantes. As gestantes realizaram seis sessões de acupuntura, com periodicidade de duas sessões por semana e duração de 30 minutos. Antes da primeira sessão, aplicou-se instrumento de coleta de dados sociodemográficos, clínicos e obstétricos. Semanalmente, foi aplicada a Escala Global de Estresse Percebido (PSS10) para verificar a evolução do estresse durante o tratamento.

**Resultados:**

Após a intervenção, houve uma diminuição significativa na pontuação dos seguintes itens da escala: preocupação, incapacidade de controle, nervosismo, cansaço, fúria e incapacidade de superar o estresse (p < 0,05). Observou-se aumento significativo na pontuação do item controle das situações (p = 0,003). Houve diferença significativa na média de estresse percebido da sessão inicial em relação às sessões da 1ª, 2ª e 3ª semana (p < 0,001).

**Conclusão:**

O uso da acupuntura para tratamento do estresse na gestação reduziu o estresse percebido pelas gestantes.

## RESUMEN

**Objetivo:**

Analizar los efectos de la acupuntura en el tratamiento de estrés percibido en embarazadas.

**Método:**

Estudio de intervención del tipo antes y después, realizado en Unidad Básica de Salud de Fortaleza, Ceará, Brasil, con 56 embarazadas. Las embarazadas realizaron seis sesiones de acupuntura, con periodicidad de dos sesiones semanales y duración de 30 minutos. Antes de la primera sesión se aplicó instrumento de recolección de datos sociodemográficos, clínicos y obstétricos. Se aplicó semanalmente la Escala Global de Estrés Percibido (PSS10) para averiguar la evolución del estrés durante el tratamiento.

**Resultados:**

Tras la intervención, hubo disminución significativa en la puntuación de los siguientes ítems de la escala: preocupación, incapacidad de control, nerviosismo, cansancio, rabia e incapacidad de superación del estrés (p < 0,05). Se observó notable crecimiento en la puntuación del ítem control de las situaciones (p = 0,003). Hubo diferencia significativa en la media de estrés percibido de la sesión inicial en relación a las sesiones de la 1ª, 2ª y 3ª semana (p < 0,001).

**Conclusión:**

El uso de la acupuntura para el tratamiento del estrés en el embarazo redujo el estrés percibido por las embarazadas.

## INTRODUCTION

Stress is an organic reaction with physical and/or psychological components, being caused by the psychophysiological changes taking place when the person faces situations of irritation, fear, or excitement^(1)^. According to the World Health Organization, stress affects 90% of the world’s population^(2)^.

During pregnancy, the woman’s body undergoes psychological and physiological changes, which can be stressor agents and trigger psychological responses that affect the maternal quality of life^(3)^. The prevalence of perceived stress among pregnant women in developing countries ranges from 11.6% to 34.2%, with a higher occurrence in the first trimester^(4,5)^.

Emotional suffering during pregnancy is influenced by both the maternal history of adversity and the stressors experienced in the prenatal period^(6)^. Among the risk factors, there is a history of domestic violence, depression, stressful life events, and interpersonal conflicts^(4)^.

The magnitude of this problem goes beyond the pregnant woman. The stress experienced during pregnancy jeopardizes the well-being of the mother, the fetus or of both, and can mean risky pregnancy that will affect not only the mother, biologically, but may also impact the baby in the social and psychological spheres^(7)^. Stress during pregnancy is a risk factor for premature birth and low birth weight^(8)^.

Furthermore, long-term intrauterine exposure to excessively secreted maternal cortisol in stressful situations during pregnancy has a negative impact on the baby’s mental health after birth. Anxiety experienced during pregnancy contributes to hyperactivity, emotional disorders, and relationship disorders in childhood^(9)^.

Given the maternal-infant effects associated with pregnancy stress, health workers shall be aware of screening this problem and using effective strategies to control it. Integrative and Complementary Practices (*PIC*) emerge as an alternative to provide relaxation, reduce bodily tensions and control stress, promoting the humanization of care and comprehensive care for women, especially during childbirth. Among them, acupuncture is highlighted^(10)^.

In Brazil, the Ministry of Health implemented the National Policy on Integrative and Complementary Practices (*PNPIC*) in the Brazilian Public Health System (*SUS*), using the term Integrative and Complementary Practices (*PIC*) to designate these approaches, which include complex medical systems and therapeutic resources^(11)^.

Acupuncture is a traditional Chinese medicine therapy method used for various purposes, including the treatment of stress-related disorders^(12)^. It has more than 2,000 years of existence and is based on the stimulation of body points using different techniques, such as needles, moxibustion, manual pressure, and direct and alternating currents, with therapeutic purposes^(13)^.

In maternal health, acupuncture has been used as an effective method to relieve complaints of nausea, vomiting, migraine, depression, and low back pain during pregnancy^(14–16)^. Moreover, it has proven effectiveness in relieving pain during childbirth^(17)^. However, further research is still required focusing on the use of acupuncture for the specific purpose of promoting stress management during pregnancy^(15,18)^. Thus, the supported hypothesis is that acupuncture has a positive effect on perceived stress in pregnant women.

Nurses play an important role in prenatal care, aiming to ensure pregnant women’s biopsychosocial well-being and to prepare them for childbirth. Acupuncture is a safe and cost-effective technique, which can be used by nurses alone or combined with other therapeutic resources in prenatal consultations, as a way of promoting relaxation and emotional control for the pregnant woman, acting in the prevention of complications related to gestational stress that can affect the mother and child in the short and long term.

The present study aims to analyze the effects of acupuncture in the treatment of perceived stress in pregnant women.

## METHOD

### Design of Study

This is a before-after intervention study.

### Local

The study was carried out in a primary health unit, located in Fortaleza-Ceará. This service offers usual-risk prenatal nursing consultations.

### Selection Criteria

The inclusion criteria were: being pregnant and followed by usual-risk prenatal care, with a gestational age between 14 and 37 weeks; presenting stress complaint; not presenting clinical or obstetric complications; not having a mental disability; not having speech or hearing problems; not having phobia of needles; being available to go to the study site for two weekly meetings. The following exclusion criteria were considered: having used analgesics in the last eight hours; and developing any risky clinical or obstetric pathology.

We chose the limit of 14 weeks for gestational age due to the beginning of the process of embryo formation and risk of miscarriage, since the stimulation of some points in the first trimester of pregnancy is contraindicated. The choice of a gestational age of less than 37 weeks was due to the likely number of acupuncture sessions required to complete the treatment of pregnant women, minimizing possible losses due to childbirth.

### Sample Definition

The population consisted of 180 women who underwent prenatal care in the aforementioned unity. Pregnant women were recruited through the convenience sampling technique. Considering the inclusion and exclusion criteria, 56 women were considered eligible. During the intervention, there were losses related to no return to the sessions and others related to obstetric complications, totaling 27 losses, with the final sample consisting of 29 pregnant women.

### Data Collection

Data collection was carried out between June and October 2016, with six acupuncture sessions for each pregnant woman, with two 30-minute sessions per week. The therapeutic intervention was performed by an obstetric nurse and acupuncturist, with five years of experience in acupuncture, working mainly in women’s health care focused on the pregnancy- puerperal cycle.

The medical records were used to identify the pregnant women seen at the service who met the inclusion criteria. Subsequently, these women were invited to participate in the study while waiting for the nursing consultation. To define the participants’ initial profile, sociodemographic, clinical, and obstetric data were collected, and the Global Perceived Stress Scale (PSS10) was applied. The scale was applied at the admission of the pregnant woman, before she entered the office (initial moment), at the 1st, 2nd, and 3rd week of follow-up, specifically in sessions 2, 4 and 6, with the SPSS10 scale being applied before the beginning of each session, based on an interview.

The Global Perceived Stress Scale (PSS10) is considered a widely applicable instrument for any subgroup of the population, including pregnant women^(19,20)^. The PSS10 scale version investigates, through 10 questions, feelings and thoughts related to stress during the past month, with six negative and four positive items. Responses range from 0 to 4 (0 = never; 1 = almost never; 2 = sometimes; 3 = almost always; 4 = always). In the present study, the PSS 10 version was adapted to investigate the feelings and thoughts of pregnant women in the past week. The scale final score can range from 0 to 40 points, and the higher the scores, the greater the stress perceived by the individual^(21)^.

Upon entering the office, the pregnant women were invited to wear specific clothing for acupuncture, lie down on the stretcher, and position themselves on the left lateral decubitus position. The needle packages were unsealed in the sight of the participant. After antisepsis with 70% alcohol, the needle was applied to the chosen points, and remained in place for 30 minutes. It should be noted that there was manipulation of the right ear, as the pregnant women remained in the left lateral decubitus position. The needle size was the same as for systemic acupuncture, 25 ṡ 30mm.

The intervention was based on the Auteroche protocol for the treatment of stress in pregnant women^(22)^.The auricular points used in the treatment were C7, VG2O, and *Yntang.* C7 has traditional energy functions capable of harmonizing the *Qi* of the heart and the *Yong Qz* ^(23)^. The VG20 point, *Bahui*, receives energy from all secondary channels from the Yang Channels of the hand and foot, maintains the *Yang Qi* of the body, removes and disperses excess *yang* of *Yang* Energy Channels, stabilizes the rise of *Yang Qi*, calms the *Shen* and emotions, clears the mind, revives unconsciousness, relaxes tendons and muscles, decreases rotational dizziness, insomnia, anxiety, palpitation, and desire to cry. At this point, it should be noted that the *Yntang* calm the mind *Shen,* being an emotional/mental harmonizer and tranquilizer^(23)^.

### Data Analysis and Treatment

Statistical analysis and crossing of variables were performed in the software *Statistical Package for the Social Science* (SPSS), version 21.0. The mean score obtained in the PSS10 scale at baseline was compared to the first, second, and third weeks of treatment. The absolute and relative frequencies were calculated for the categorical variables, as well as the mean and standard deviation for the numerical variables.

To evaluate the mean difference in the four periods, a test of Repeated Measures Analysis of Variance (ANOVA-MR) was performed. To apply the test, initially the sphericity test of variances of the moments was carried out, considering p > 0.05 as a homogeneous analysis. The difference in means between all periods was evaluated considering p value <0.05 as statistically significant. Finally, the effect size was evaluated by the Partial Eta Squared (η^2^).

### Ethical Aspects

The study was approved by the Research Ethics Committee of the Maternidade Escola Assis Chateaubriand, under Opinion 1.553.641, with approval in 2016. The study followed the ethical recommendations for research with human beings, according to Resolution No. 466/2021 of the National Health Council. The pregnant women were invited to participate in the study, and, later, the Free and Informed Consent Form (FICF) was read together with the researcher, and then signed.

## RESULTS

**Figure 1.**
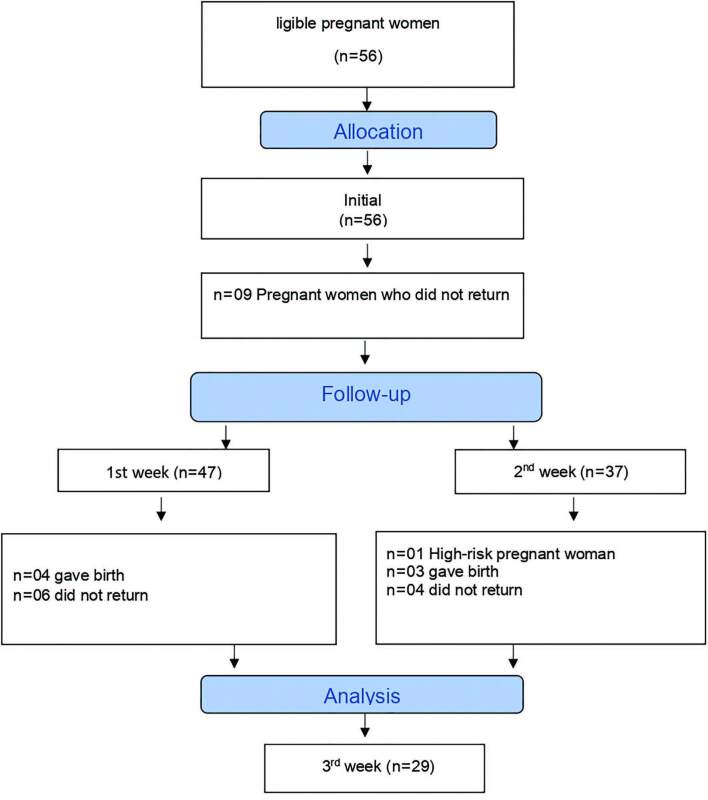
Flowchart of data collection and follow-up losses.

Of the 56 pregnant women who started participating in the study, 9 dropped out after the first week. Between the 2nd and 3rd weeks of follow-up, there were 10 dropouts. Between the 3rd week and the end of the study, there were 8 losses, making a sample of 29 participants who completed the study, Figure 1.

Most pregnant women were between 20 and 29 years old (58.9%), with a mean of 26 ± 6.44 years. Regarding education, 44.6% had completed high school, with a mean value of 12 ± 2.68 years of study. There was a predominance of pregnant women residing in Fortaleza-Ceará (96.5%). As for income, 46.6% received one to two minimum wages. Most pregnant women reported living with a partner (80.4%) and there was a predominance of housewives (44.6%).

Regarding gestational age, 42.8% of pregnant women were in the second trimester and 57.2% were in the third trimester. As for the number of deliveries, 55.4% were primiparous. Regarding physical activity, only 14.3% of pregnant women reported performing some physical activity, as shown in Table 1.

**Table 1.** Sociodemographic and clinical characterization of pregnant women (n = 56) – Fortaleza, CE, Brazil, 2016.

Variables	N	%
**Age range**		
Up to 19 years	08	14.3
From 20 to 29 years old	33	58.9
30 years old or more	15	26.8
**Level of education**		
Up to 09 years	13	23.2
From 10 to 12 years	25	44.6
13 years old or more	18	32.2
**Origin**		
Capital	54	96.5
Inland city	02	3.5
**Income (in minimum wages)**		
Up to 1	01	1.8
Between 1–2	25	44.6
Between 2–3	18	32.2
Between 3–4	06	10.7
More than 4	06	10.7
**Type of union**		
With partner	45	80.4
With no partner	11	19.6
**Occupation**		
Housewife	25	44.6
Work out	07	12.5
Student	07	12.5
Both	17	30.4
**Gestational Age**		
Second trimester	24	42.8
Third trimester	32	57.2
**Parity**		
Primiparous	31	55.4
Multiparous	25	44.6
**Carrying out physical activity**		
Yes	8	14.3
No	48	85.7

As observed on Table 2, after the intervention, there was a significant decrease in the scores of the following scale items: being upset, inability to control, nervousness, tiredness, anger, and inability to overcome stress. In addition, there was a statistically significant increase in the score for the item control of situations.

**Table 2.** Mean score and standard deviation of PSS10 scale items, according to type of alteration, measurement moment, and p-value (n = 29) – Fortaleza, CE, Brazil, 2016.

Stress scale questions	Initial	1st week	2nd week	3rd week	p-value*
**Items that decreased**					
(P1) Worries	2.22 ± 1.33	1.41 ± 1.33	1.59 ± 1.19	1.42 ± 1.29	**0.002**
P2: Inability to control	2.28 ± 1.31	1.36 ± 1.17	1.30 ± 1.17	1.55 ± 1.05	**<0.001**
P3: Nervousness	3.05 ± 0.98	1.93 ± 1.31	1.70 ± 1.11	1.62 ± 1.01	**<0.001**
P6: Tiredness	2.44 ± 1.11	1.71 ± 1.17	1.67 ± 1.25	1.27 ± 1.13	**<0.001**
P9: Anger	2.41 ± 1.29	1.73 ± 1.45	1.67 ± 1.44	1.27 ± 1.31	**<0.001**
P10: Inability to overcome stress	1.91 ± 1.35	1.47 ± 1.32	1.19 ± 1.15	0.86 ± 1.22	**<0.001**
**Items that have not changed**					
P5: Capacity to control	2.04 ± 1.32	2.38 ± 1.33	2.11 ± 1.17	2.04 ± 1.08	0.538
**Items that increased**					
P8: Control of situations	1.46 ± 1.14	2.24 ± 1.13	2.22 ± 1.03	1.93 ± 1.19	**0.003**
P7: Irritation control	2.02 ± 1.42	2.25 ± 1.36	2.43 ± 1.14	2.04 ± 1.27	0.361
P4: Confidence to face the problem	2.57 ± 1.23	2.68 ± 1.33	2.76 ± 1.09	2.65 ± 1.39	0.754

*RM-ANOVA test.

Regarding PSS10 scale total score, it was observed that the mean perceived stress in the initial assessment was 22.4 ± 5.0. In the first week of follow-up, the observed mean of the scale was 19.1 ± 5.1, in the second week it was 18.2 ± 6.9, and in the third week it was 16.7 ± 6.7. Thus, an RM-ANOVA was performed to assess the difference in scale scores at baseline and follow-ups in the first, second, and third weeks. Mauchly’s sphericity test followed the sphericity assumption (Mauchly’s W = 0.735; χ^2^ **(**5) = 8.24; p = 0.144). The overall result of RM-ANOVA showed a statistically significant difference in perceived stress over the four periods (F(3.84) = 14.46; p < 0.001; η^2^= 0.341) (Table 3).

**Table 3.** Assessment of the change in the average trend of perceived stress over time (n = 29) – Fortaleza, CE, Brazil, 2016.

Reference period	Comparison period	Difference of Means	Standard error	p-value*	95% CI for the difference in means	
					Lower limit	Upper limit
Initial	1st week	3.21	0.79	0.002	0.97	5.45
	2nd week	3.70	0.99	0.005	0.88	6.50
	3rd week	6.14	1.08	<0.001	3.06	9.21
1st week	2nd week	0.48	0.72	1.000	–1.55	2.52
	3rd week	2.93	1.02	0.046	0.04	5.82
2nd week	3rd week	2.45	0.98	0.110	–.33	5.22

*RM-ANOVA test.

In addition, a posteriori analysis (*post hoc* Bonferroni) showed that there was a significant decrease in perceived stress levels at the three-week follow-up compared to the initial assessment. However, the comparisons showed a possible loss of effect over time, since there is a relationship, at the limit of statistical significance, between the first and third weeks; and the loss of association between the first and second weeks of follow-up and between the second and third weeks. This fact may be related to the sample loss over time (Table 3).

## DISCUSSION

According to the sample sociodemographic profile, pregnant women’s mean age was similar to data from another study, whose mean age was 26 years^(24)^. Corroborating the present study, another national survey showed that pregnant women’s education years ranged between 9 and 12 years^(25)^. The level of education should be considered in the prenatal consultation, as it can influence the understanding of the information provided during follow-up^(24)^. Thus, women with less education have low adequacy to prenatal care, less access to information, and limited health care.

Regarding the obstetric profile, it was found that there was a predominance of gestational age in the third trimester and of primiparous women. Probably, the experience of becoming pregnant for the first time and the proximity of delivery influenced the pregnant women’s perception of stress and, consequently, the decision to accept acupuncture treatment.

The present study showed a reduction in perceived stress after six acupuncture sessions. A similar study, however, with different acupressure points, carried out with pregnant women in the United States, also had a significant reduction in stress^(26)^. The progressive improvement in the stress perceived by the pregnant women throughout the sessions converges with the results obtained in a Brazilian case report, in which there was a partial decrease in anxiety symptoms from the fourth session and a significant improvement of the pregnant woman, with a report of symptoms relief, from the sixth treatment session on^(27)^.

The effectiveness of acupuncture in treating stress in pregnant women is also in line with the results of a systematic review analyzing five clinical trials that showed a greater overall reduction in anxiety and stress in the acupressure group than in sham controls^(28)^.

Acupuncture has been evidenced in general treatment, gaining space and acceptance in the scientific environment^(27–29)^. Among the advantages of this technique, the relatively low cost, practicality, and limited possibility of adverse events are highlighted^(30)^.

Given the hormonal reactions and variations in blood flow in the uterus caused by stress, which directly influence the intrauterine environment and the psychological relationship between mother and fetus^(9)^, the application of acupuncture therapy for the promotion of pregnant women’s mental health and consequent repercussion towards the healthy development of the concept becomes promising^(28)^. Since the points used in the study are to stimulate the circulation of the *Qi* and *Xue*, these, when stimulated, reorganize the whole body energy circulation, thus restoring the balance between opposing status of meridian function^(28)^.

Regarding the risks to the child’s health, a study of human observations indicated that stressful experiences during pregnancy are associated with an increased risk of schizophrenia in children^(9)^. In addition, stress in pregnancy can increase the risk of psychiatric disorders, attention deficit and hyperactivity disorder, anxiety, and delay in language use in children^(9)^.

In view of the risks of stress for maternal and fetal morbidity, as well as the proven benefits of using acupuncture during pregnancy to control perceived stress, the possibility of nurses who provide care to women during prenatal care to use non-pharmacological strategies to promote emotional control, providing comprehensive and humanized care, is emphasized. As specific qualification is required for the practice of acupuncture by health professionals, there is the possibility of referring patients from the *PIC*s to services that offer care.

As limitations of the present study, we cite the short follow-up time, the loss of follow-up, and the absence of a control group, which limit the possibility of generalizing the results and the strength of the evidence found. These conditions can be considered when conducting future research. It should be noted that only the protocol indicated in acupuncture for the treatment of stress in pregnant women was used; therefore, caution is advised when generalizing the results found.

## CONCLUSION

The study showed a positive effect of acupuncture sessions follow-up on the reduction of perceived stress, with significant improvement in perceived stress with each session performed and significant decrease in perceived stress levels at three weeks of follow-up compared to baseline. Based on these results, acupuncture can be recommended as a fundamental resource for interventions aimed at promoting pregnant women’s mental health in usual-risk prenatal care. However, experimental studies capable of controlling the variables that may interfere with perceived stress, to better elucidate the stress perceived by pregnant women, are required.

## ASSOCIATE EDITOR

Maria Luiza Gonzalez Riesco
